# Alginate–Bentonite Beads Containing Thiamethoxam: Preparation, Characterization, and Activity against *Brevicoryne brassicae*

**DOI:** 10.1021/acsomega.6c06825

**Published:** 2026-09-17

**Authors:** Silas J. de Souza, Gustavo F. de Castro, Jader A. Ferreira, Renê C. da Silva, Carlos G. da Cruz, Flávio L. Fernandes, Jairo Tronto

**Affiliations:** † Instituto de Ciências Exatas e Tecnológicas, 28120 Universidade Federal de Viçosa—UFV, Rio Paranaíba, Minas Gerais 38810-000, Brazil; ‡ Departamento de Agronomia, Universidade Federal de Viçosa—UFV, Viçosa, Minas Gerais 36570-900, Brazil; § Instituto de Ciências Agrárias, Universidade Federal de Viçosa—UFV, Rio Paranaíba, Minas Gerais 38810-000, Brazil

## Abstract

Brevicoryne brassicae is an important pest of brassica crops, and thiamethoxam is widely used for its control. This study prepared alginate–bentonite beads containing thiamethoxam and evaluated their physicochemical characteristics, aqueous recovery profile, soil-column leachate profile, and insecticidal activity against *B. brassicae*. X-ray diffraction, ATR-FTIR, TGA–DTG, and scanning electron microscopy characterized the matrix, while HPLC established an extractable thiamethoxam content of 1.219 ± 0.049 mg g^–1^. In the aqueous assay, the fraction recovered from the medium was lower for the bead formulation than for Actara 250 WG over the documented 48 h interval. Recalculation of the soil-column records gave cumulative fractions of 81.13 ± 1.12% for the bead formulation and 94.15 ± 6.91% for Actara 250 WG on day 30 (*n* = 4). The bead treatment produced high mean mortality of *B. brassicae* at the evaluated challenge times. However, the formulations were not applied at equivalent thiamethoxam amounts, and the experiments did not include a complete soil-column mass balance or formal equivalence or noninferiority analyses. The results therefore demonstrate different behavior under the tested conditions but do not establish superior performance, prolonged insect control, groundwater protection, or reduced environmental impact.

## Graphical abstract

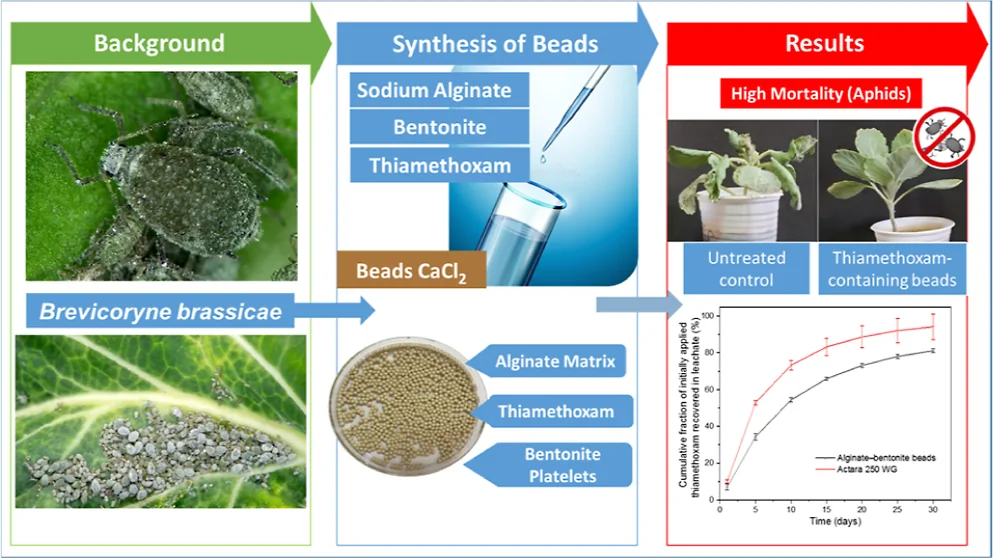

## 1 Introduction

According to the United Nations, the global population is projected to increase by approximately 2 billion people over the next 30 years, reaching 9.7 billion by 2050.^1^ This population growth is expected to substantially increase global food demand. However, meeting this growing demand may intensify several challenges, including the excessive use of pesticides to control pests and reduce agricultural losses.^2–4^

The widespread use of pesticides began in the 1950s during the so-called “Green Revolution,” which, according to its proponents, aimed to combat global hunger through the adoption of new agricultural practices.^5^ Although pesticides have played an essential role in increasing food production and reducing crop losses,^4^ their intensive use has also resulted in adverse effects on human health, including headaches, diabetes, neurodegenerative diseases, and different types of cancer, as well as environmental impacts such as contamination of water, soil, and air, degradation of soil microbial functions, and the selection of resistant pest populations.^6–8^

Cabbage (*Brassica oleracea* var. capitata) is an economically important crop, with annual global production exceeding 72 million tons. By 2024, the cabbage market is expected to reach USD 47.08 billion, with Asia-Pacific countries representing the largest producers and China leading global production and exports.^9^ However, the cabbage aphid, *Brevicoryne brassicae* (L.) (Hemiptera: Aphididae), is one of the most destructive pests affecting brassica crops worldwide,^10–12^ causing yield losses of up to 82% in certain genotypes.^11–13^

Chemical control using insecticides and plant extracts remains a commonly adopted strategy for managing cabbage aphid populations.^14^ Among the insecticides used for this purpose, thiamethoxam is one of the most widely used.^15^ Thiamethoxam is a group 4A neonicotinoid that acts as a competitive modulator of insect nicotinic acetylcholine receptors. Binding to the acetylcholine site disrupts cholinergic neurotransmission and may cause hyperexcitation, lethargy, paralysis, and death. Thiamethoxam is not an acetylcholinesterase inhibitor.^16^ According to the current Brazilian product label, Actara 250 WG contains 250 g kg^–1^ thiamethoxam and is classified as Toxicological Category 5 and Environmental Hazard Class III.^17^

Given the environmental and health concerns associated with conventional pesticide use, developing alternative approaches capable of maintaining agricultural productivity while minimizing harmful effects has become increasingly important. In this context, organic–inorganic hybrid materials have emerged as promising alternatives due to their physicochemical properties and versatility. These materials have been applied in agriculture to improve plant germination and growth, enable the controlled release of nutrients and pesticides, and enhance overall crop productivity.^18,19^

Biodegradable natural polymers such as alginate are commonly used in the preparation of hybrid materials for controlled-release agricultural formulations.^20,21^ Alginate, a low-cost and nontoxic biopolymer obtained from brown algae, has attracted considerable attention due to its water retention capacity, biodegradability, viscosity, and ability to form gels in the presence of cross-linking agents.

Bentonite, a clay composed mainly of montmorillonite, is a highly versatile material used in hybrid formulations. Characteristics such as high specific surface area, cation exchange capacity, and moderate negative surface charge make bentonite valuable for several industrial applications, ranging from waterproofing materials to cosmetics and pharmaceutical products.^22^

Recent nanoenabled and carrier-based pest-management strategies have employed engineered material architectures to regulate the transport, availability, and persistence of active agents or semiochemicals. For example, graphene oxide-based interlayer systems have been used to extend the diffusion pathway and effective lifetime of a pheromone applied in targeted pest control. Such studies illustrate how carrier architecture can influence the delivery of pest-management agents. The alginate–bentonite carriers evaluated in the present study, however, are millimeter-scale beads and are not classified as nanoparticles.^23^

Alginate–bentonite carrier systems may alter the aqueous transport and soil-column leachate profile of an active ingredient through the properties of the cross-linked polymer–clay matrix. However, the specific association mode and transport mechanism must be established experimentally for each formulation. To the best of our knowledge, alginate–bentonite millimeter-scale beads containing thiamethoxam have not been evaluated against *B. brassicae*. The present study therefore examines the preparation and behavior of this formulation without presuming a demonstrated controlled-release mechanism or superior environmental performance.

Therefore, this study aimed to (i) prepare and characterize alginate–bentonite beads containing thiamethoxam; (ii) determine their extractable thiamethoxam content by HPLC; (iii) compare the fraction of initially applied thiamethoxam recovered from the aqueous medium for the bead formulation and Actara 250 WG; (iv) evaluate the cumulative fraction recovered from soil-column leachate; and (v) assess mean insecticidal activity against *B. brassicae* at the evaluated challenge times.

## 2 Material and Methods

### 2.1 Preparation of Beads

#### 2.1.1 Preparation of Control Beads

The control beads were prepared using the complex coacervation method, adapted from Zhang et al.^24^ A gel was prepared with 50 mL of water and 0.5 g of sodium alginate and stirred for approximately 1 h at 25 °C. Subsequently, 2.5 g of bentonite was dispersed in the gel and the suspension was stirred for another 1 h. The suspension was transferred to a 50 mL buret and added at approximately one drop per second to a 5% (m/v) CaCl_2_ solution. The resulting beads were sieved to remove excess solution, transferred to a Petri dish, and dried in a forced-air oven for approximately 24 h at 40 °C. The dried beads were placed in a closed dry container and stored under refrigeration until characterization and bioassay use. Photographs of the control beads before and after drying are provided as Figure S1 in the Supporting Information.

#### 2.1.2 Preparation of Thiamethoxam-Containing Beads

The thiamethoxam-containing beads were prepared using the same complex coacervation procedure. A 1000 mg L^–1^ thiamethoxam stock solution was diluted to 350 mg L^–1^ in a final volume of 50 mL, corresponding to 17.5 mg of initially added thiamethoxam. Sodium alginate (0.5 g) was dissolved in this solution with stirring for 1 h at 25 °C, after which 2.5 g of sodium bentonite was added and the suspension was stirred for another 1 h. The suspension was added dropwise to a 5% (m/v) CaCl_2_ solution, and the resulting beads were dried for 24 h at 40 °C, placed in a closed dry container, and stored under refrigeration. No coupling reagent or chemical reaction designed to covalently bind thiamethoxam to alginate was used. Photographs of the thiamethoxam-containing beads before and after drying are provided as Figure S2 in the Supporting Information.

### 2.2 Characterization of Materials

For X-ray diffraction (XRD) analyses, the samples were previously macerated in an agate mortar to obtain particles of uniform size. To ensure consistency across all XRD analyses, 0.5 g of each sample in powder form was placed in an aluminum sample holder. XRD analyses were performed on a Shimadzu XRD-6000 diffractometer equipped with a Cu cathode and a graphite crystal monochromator to select the Cu Kα_1_ emission region (λ = 1.5406 Å). The diffraction patterns were recorded with a step size of 0.02°/s over a 2θ range of 4°–70°.

Attenuated total reflectance Fourier transform infrared spectroscopy (ATR-FTIR) spectra were recorded on a Jasco 4100 spectrometer using 256 scans, with a resolution of 4 cm^–1^ over a wavenumber range of 4000–400 cm^–1^.

Thermogravimetric analysis and derivative thermogravimetry (TGA–DTG) were performed using a TGA Q500 instrument (TA Instruments, USA). The heating rate was 10 °C min^–1^ under an O_2_ flow of 50 cm^3^ min^–1^, from room temperature to 1000 °C. Sample masses ranged from 2.0 to 4.0 mg.

Scanning electron microscopy (SEM) images were obtained using a Carl Zeiss EVO 50 microscope. The samples were mounted on a sample holder using conductive double-sided adhesive tape.

To determine the extractable thiamethoxam content, approximately 0.06 g of dried thiamethoxam-containing beads was extracted in 100 mL of water for 96 h at 25 °C and 110 rpm. A 1.0 mL aliquot was filtered through a 0.45 μm syringe filter and analyzed using an Agilent 1260 Infinity II HPLC equipped with a UV detector at 225 nm and a C18 column (Luna Phenomenex, 4.6 mm × 250 mm × 5 μm). The mobile phase was 0.01 mol L^–1^ H_3_PO_4_ aqueous solution/acetonitrile (70:30, v/v), the column temperature was 35 °C, the flow rate was 1.0 mL min^–1^, and the injection volume was 20 μL. The low-concentration calibration series comprised 0, 0.05, 0.10, 0.20, 0.30, 0.40, 0.50, 0.60, 0.70, 0.80, 0.90, and 1.00 mg L^–1^ and gave *y* = 335.6379193*x* + 0.244129 (*R* ^2^ = 0.9999479185), where y is chromatographic peak area and x is thiamethoxam concentration in mg L^–1^. The archived data set comprised six analytical determinations arranged in two triplicate sets.

The concentration of thiamethoxam in the extract was calculated from the external calibration curve according to the following equation

C=(y−0.244129)/335.6379193

where *C* is the thiamethoxam concentration in the extract (mg L^–1^) and *y* is the chromatographic peak area.

The extractable thiamethoxam content in the beads (*C*_ext_, mg g^–1^) was calculated using the following equation

$$C_{ext}=\left(C\times V_{ext}\right)/m_{bead}$$

where *C* is the thiamethoxam concentration in the extract (mg L^–1^), *V*_ext_ is the extraction volume (L), and *m*_bead_ is the dry mass of the extracted beads (g).

The extractable thiamethoxam content of the dried alginate–bentonite beads was 1.219 ± 0.049 mg g^–1^, based on six analytical determinations arranged in two triplicate sets (RSD = 4.06%). The analytical procedure was not evaluated using thiamethoxam-free beads fortified before extraction. Therefore, the reported value was not corrected for matrix-specific analytical recovery and is described as extractable thiamethoxam content rather than as a validated total-content measurement. The total dry mass recovered from each preparation batch and the thiamethoxam contents of the CaCl_2_ bath and processing liquids were not recorded. Consequently, material yield, conventional encapsulation efficiency, and process losses could not be reliably reconstructed retrospectively.

### 2.3 Aqueous Thiamethoxam-Recovery Assay

The aqueous assay was conducted in phosphate buffer at pH 6.0. The buffer was prepared by mixing 500 mL of 0.2 mol L^–1^ KH_2_PO_4_ with 57 mL of 0.2 mol L^–1^ NaOH and adjusting the final volume to 2.0 L with water. The pH was adjusted during buffer preparation using HNO_3_ or NaOH and measured with a QUIMIS Q400AS pH meter. A phosphate buffer at pH 6.0 was selected to provide a controlled aqueous condition close to the pH of the soil used in the column experiment, which had a measured pH in water of 6.05.

Three treatments were evaluated: T1, control beads without thiamethoxam; T2, thiamethoxam-containing beads; and T3, Actara 250 WG. The archived records are consistent with a destructive-sampling design using a separate set of three responses for each treatment and sampling time. Actual formulation masses were 0.0508–0.0586 g for the bead formulation and 0.0101–0.0114 g for Actara 250 WG (Table 1). Each vessel contained 100 mL of phosphate buffer and was maintained at 25 °C and 110 rpm. Samples were collected at 0, 0.25, 0.50, 0.75, 1, 2, 3, 4, 5, 6, 7, 8, 9, 10, 11, 12, 24, and 48 h. A 1.0 mL aliquot was collected and filtered, after which the remaining medium in the sampled vessel was discarded. Because flask-specific identifiers were not retained, the three values at each time are described as archived replicate responses. Entries at 72, 96, and 120 h were not included because their status as a validated continuation of the documented experiment could not be established.

**Table 1:** Amounts of Bead Formulation and Actara 250 WG Used in Each Experiment and Their Corresponding Extractable and Nominal Thiamethoxam Contentes

experiment	bead formulation	calculated extractable THX in beads	Actara 250 WG	nominal THX in Actara
aqueous assay	0.0508–0.0586 g per flask	0.0619–0.0714 mg per flask	0.0101–0.0114 g per flask	2.525–2.850 mg per flask
soil-column assay	1.0038–1.0090 g per column	1.224–1.230 mg per column	0.0100–0.0109 g per column	2.500–2.725 mg per column
bioassay	4330 g formulation ha^–1^	5.28 g extractable THX ha^–1^	200 g formulation ha^–1^	50.0 g nominal THX ha^–1^

The cumulative percentage of thiamethoxam released at each sampling time was calculated as

$$P_{t,r}\left(\%\right)=\left(C_{t,r}\times V/M_{0,t,r}\right)\times 100$$

where *P*_t,r_ is the unadjusted percentage of thiamethoxam released (%) for replicate *r* at sampling time *t*, *C*_t,r_ is the thiamethoxam concentration determined by HPLC (mg L^–1^), *V* is the total volume of the release medium (L), and *M*_0,t,r_ is the calculated extractable thiamethoxam content corresponding to the amount of bead formulation used in replicate *r* (mg).To correct for the initial amount of thiamethoxam released at time zero, the release percentages were baseline-adjusted using the following equation

$$P_{t,adj}\left(\%\right)={\mathrm{P̅}}_{t}-{\mathrm{P̅}}_{0}$$

where *P*_t,adj_ is the baseline-adjusted percentage of thiamethoxam released (%), *P̅*_*t*_ is the mean unadjusted release percentage at sampling time *t*, and *P̅*_0_ is the mean unadjusted release percentage at time zero. The standard deviation for each sampling time was calculated from the three archived replicate measurements.

For the beads, *M*_0_ was calculated from the actual bead mass and the measured extractable content of 1.218621605 mg g^–1^; for Actara 250 WG, *M*_0_ was calculated from the actual formulation mass and the nominal content of 250 mg g^–1^. Because each sampled vessel was discarded, the reported values are not a cumulative sum of serial aliquots. No kinetic or mechanistic release model was fitted.

### 2.4 Leaching Test in Soil Columns

The soil sample used in this test was collected in Rio Paranaíba, Minas Gerais State, Brazil (latitude: −19.1942, longitude: −46.2437). The soil exhibited the following physical and chemical properties: clay 781 g kg^–1^; silt 131 g kg^–1^; fine sand 54 g kg^–1^; coarse sand 35 g kg^–1^; soil bulk density 1.12 g cm^–3^; total porosity 0.49 m^3^ m^–3^; pH (H_2_O) = 6.05; P 0.8 mg dm^–3^; K 45 mg dm^–3^; Ca^2+^ 1.51 cmolc dm^–3^; Mg^2+^ 0.55 cmolc dm^–3^; Al^3+^ 0 cmolc dm^–3^; H^+^ + Al^3+^ 3.1 cmolc dm^–3^; cation exchange capacity 2.18 cmolc dm^–3^; effective cation exchange capacity 2.18 cmolc dm^–3^; potential cation exchange capacity 5.28 cmolc dm^–3^; base saturation 41.3%; aluminum saturation 0%; total organic carbon 1.9%; and organic matter 3.29%. According to the Brazilian Soil Classification System, the soil is very clayey and can be classified as a dystrophic red latosol.^25^

The leaching test was adapted from the method described by Abat et al.^26^ First, 60 mL syringes (serving as columns), with a diameter of 31 mm and height of 17.5 cm, were filled with glass wool at the base to ensure the filtered solution remained clear. Next, 50 cm^3^ of previously homogenized, air-dried soil sieved to <2 mm was added. The amount of soil used in each column was determined by calculating soil porosity, as described by Teixeira et al.^27^ To settle the soil in the columns, 25 mL of water, corresponding to the soil pore volume, was added and drawn up with the syringe. Afterward, a central perforation of approximately 5 cm was made, where the microspheres were introduced. Finally, another layer of glass wool was added to seal the system.

Four treatments were evaluated: T1, control beads without thiamethoxam; T2, thiamethoxam-containing beads; T3, Actara 250 WG; and T4, untreated control. Individual bead masses ranged from 1.0038 to 1.0090 g per column, and individual Actara 250 WG masses ranged from 0.0100 to 0.0109 g per column (Table 1). Four replicate columns were included in the final analysis (*n* = 4). At 1, 5, 10, 15, 20, 25, and 30 days, 25 mL of water was added to each column, and the recovered leachate volume was measured individually. The experimental setup is shown in Figure S3 of the Supporting Information.

Leachate concentrations were recalculated consistently using the archived 0–100 mg L^–1^ calibration curve, *y* = 352.7770*x* – 66.3475 (*R* ^2^ = 0.999978), where *y* is chromatographic area and *x* is thiamethoxam concentration in mg L^–1^. The appropriate archived control and blank corrections were applied to each collection event. Some calculated concentrations were below the lowest nonzero calibration standard of 5 mg L^–1^ and were therefore treated as an analytical limitation. The cumulative fraction recovered up to collection event *j* was calculated independently for each column as

$$F_{j}\left(\%\right)=\left[\sum_{i=1}^{j}\left(C_{i}\times V_{i}\right)/M_{0}\right]\times 100$$

*C*_*i*_ = thiamethoxam concentration measured at sampling event *i*; *V_i_* = recovered volume of the medium at sampling event *i*; *M*_0_ = initial amount of thiamethoxam represented by the formulation applied in each column.

For the bead formulation, *M*_0_ was calculated from the individual bead mass and the measured extractable thiamethoxam content of 1.219 mg g^–1^. For Actara 250 WG, M_0_ was calculated from the individual formulation mass and the nominal content of 250 mg g^–1^. Results are reported as means ± sample standard deviations for four replicate columns (*n* = 4).

### 2.5 Collection and Rearing of Insects

Apterous insects of *B. brassicae* were collected from organic plantings of kale (*Brassica oleracea* L.) and cabbage (*Brassica oleracea* L. var. capitata) (Brassicaceae) in the municipality of Rio Paranaíba, Minas Gerais State, Brazil (latitude: −19.1942, longitude: −46.2437). The collections were carried out to establish a colony on cabbage leaves. During collection, the leaves of these plants containing aphids were removed and placed in plastic bags until transport to the rearing site. Aphids were reared on cabbage plants in 50 × 50 × 50 cm cages covered with an antiaphid screen. To this end, reproduction was monitored to select first-instar nymphs.

### 2.6 Bioassay

The bioassay was conducted under controlled conditions at 23–26 °C with a 12 h photoperiod. Atlanta hybrid cabbage seedlings were transplanted into 250 mL pots and treated 5 days later. The completely randomized design comprised four treatments and six replicates: beads without thiamethoxam; untreated control; thiamethoxam-containing beads at 4330 g formulation ha^–1^, corresponding to approximately 5.28 g extractable thiamethoxam ha^–1^; and Actara 250 WG at 200 g formulation ha^–1^, corresponding to 50.0 g nominal thiamethoxam ha^–1^ (Table 1). Each plant constituted an experimental unit. The treatments were not applied at equivalent thiamethoxam rates, and the bead treatment required a substantially greater formulation mass. The archived records do not preserve the treated surface area or the conversion used to determine the exact formulation mass per 250 mL pot; therefore, dose per pot was not reconstructed.

The plants received the soil treatment once and were challenged with 50 first-instar nymphs immediately after treatment application and again at 7 and 14 days. Mortality was assessed 24 h after each challenge. The archived protocol does not document whether any plants were replaced or how surviving insects were handled before each subsequent challenge. Aphids were considered dead when they did not respond to gentle probing with a brush. Corrected mortality was calculated using Abbott’s equation. Mean mortality in the untreated control was 0% at all three assessment dates; consequently, Abbott correction did not change the treatment mean values.^28^

$$Corrected\;mortality\left(\%\right)=\left[\left(M_{t}-M_{c}\right)/\left(100-M_{c}\right)\right]\times 100$$

where *M*_t_ = observed mortality in the treatment (%); *M*_c_ = observed mortality in the untreated control (%).

Treatment effects were analyzed separately at each assessment date using one-way analysis of variance followed by Tukey’s test at *p* < 0.05. These analyses were restricted to treatment comparisons within each date. No repeated-measures, treatment-by-time, equivalence, or noninferiority analysis was performed. Statistical analyses were performed using the SPEED Stat 2.4 software.^29^

## 3 Results and Discussion

X-ray diffraction (XRD) patterns of sodium alginate, sodium bentonite, Actara 250 WG, control beads, and thiamethoxam-containing beads are presented in Figure 1. The XRD pattern of the biopolymer shown in Figure 1a indicates an amorphous morphology, as evidenced by the elevated baseline and broadening of diffraction peaks. Several studies in the literature report the presence of three prominent characteristic peaks of sodium alginate at 2θ = 14°, 21°, and 38°.^30–33^

**Figure 1:**
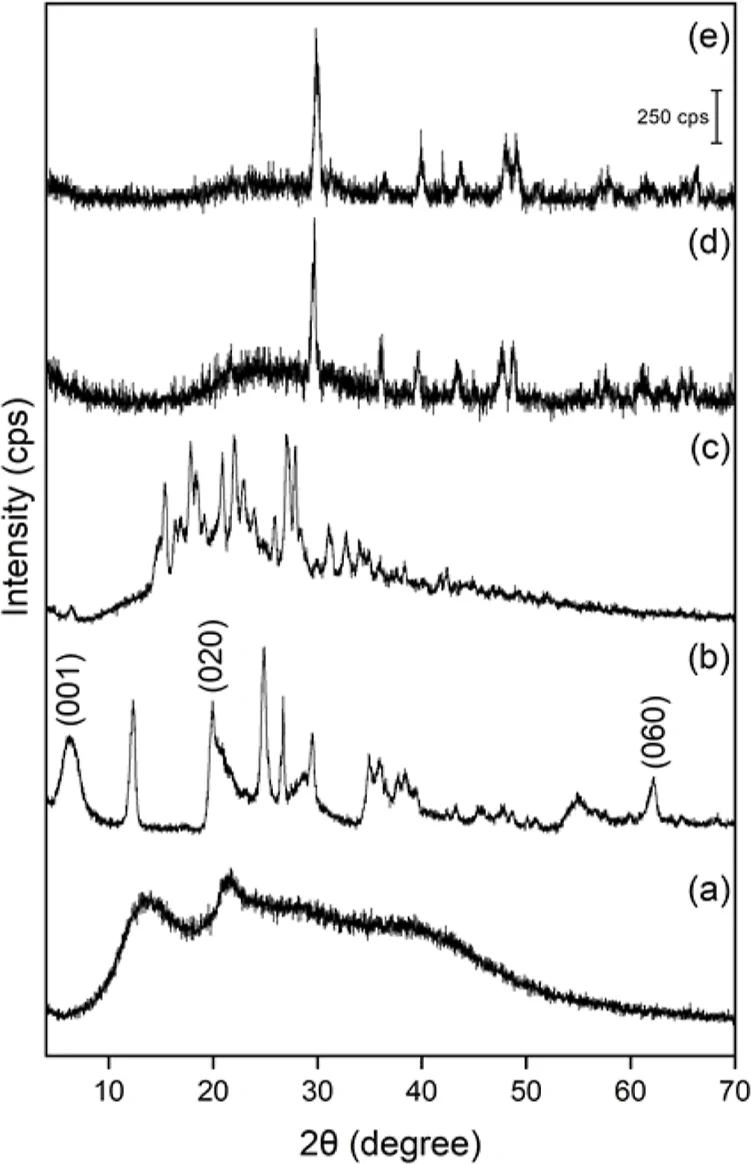
Diffraction patterns of (a) sodium alginate, (b) sodium bentonite, (c) Actara 250 WG, (d) control beads, and (e) thiamethoxam-containing beads.

The XRD pattern of sodium bentonite shown in Figure 1b displays characteristic peaks of montmorillonite, which are observed at 2θ = 6.2° (d_001_) and 19.9° (d_020_). The interplanar distances were 14.3 Å and 4.53 Å, respectively; these results are similar to those found in the literature.^34,35^ The octahedral structure of sodium bentonite is attributed to the peak observed at 2θ = 62.2° (d_060_).^34^ The other peaks observed in the XRD pattern may be related to impurities in the material, such as quartz and feldspar.

Considering the nominal initial dry-solid composition, bentonite was the major component used in bead preparation. However, the bead diffractograms showed broad and attenuated features, and no quantitative phase analysis was performed.^36^ Relative peak intensities were therefore not used to infer component proportions. No new reflection or reliable basal-spacing shift attributable specifically to thiamethoxam was identified, and the similar patterns of the control and thiamethoxam-containing beads do not demonstrate thiamethoxam intercalation or structural incorporation.

The ATR-FTIR spectra of sodium alginate, sodium bentonite, thiamethoxam standard, commercial thiamethoxam formulation, control microspheres, and thiamethoxam-loaded microspheres are shown in Figure 2. In the spectrum of sodium alginate presented in Figure 2a, the band observed at 1020 cm^–1^ is associated with the stretching vibration of the C–O–C groups. The bands at 1600 cm^–1^ and 1420 cm^–1^ are attributed to the symmetric and asymmetric stretching of the COO^–^ groups in the carboxylate structure of alginate. Lastly, the broad band at 3380 cm^–1^ corresponds to the vibrational mode of the O–H bond.^37,38^

**Figure 2:**
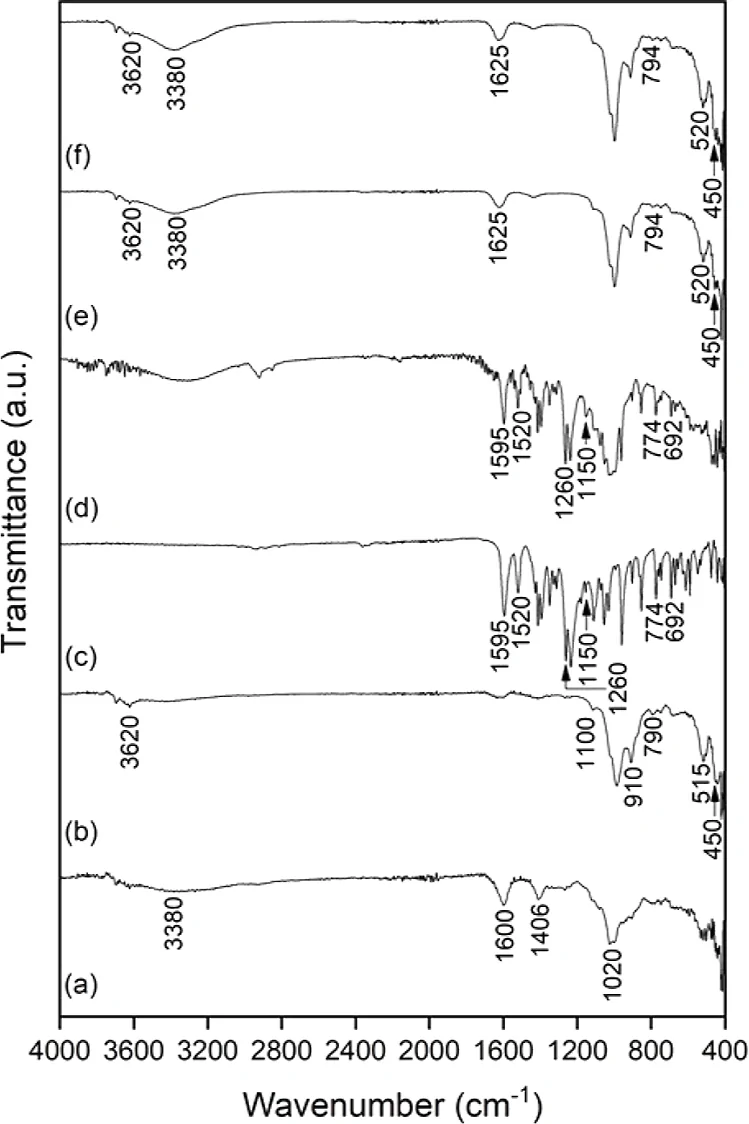
Attenuated total reflectance Fourier transform infrared spectroscopy spectra of sodium alginate (a); sodium bentonite (b); thiamethoxam standard (c); Actara 250 WG (d); control beads (e); and thiamethoxam-containing beads (f).

The ATR-FTIR spectrum of sodium bentonite, shown in Figure 2b, displays bands corresponding to O–H stretching vibrations in the region of 3620 cm^–1^. The band around 1100 cm^–1^ is attributed to the Si–O asymmetric stretching of the tetrahedral and octahedral sheets. The band at 905 cm^–1^ corresponds to the deformation vibrations of Al–Al–OH bonds. Additionally, bands at 790 cm^–1^, 515 cm^–1^, and 450 cm^–1^ are associated with the vibrations of the Si–O–Si and Si–O–Al bonds, respectively.^39,40^

Figure 2c,d show the spectra of the thiamethoxam standard and the commercial thiamethoxam formulation, respectively. Both spectra display the same characteristic bands, as expected, since thiamethoxam is the active ingredient in the commercial formulation. The band at 1595 cm^–1^ corresponds to the axial stretching of the C=C bond. The bands at 1520 cm^–1^ and 1260 cm^–1^ are attributed to the asymmetric and symmetric stretching vibrations of the NO_2_ group. The band in the region of 1150 cm^–1^ represents the asymmetric axial stretching of the C–O–C bond. The axial stretching of the alkyl halide (C–Cl) is observed at 774 cm^–1^, and the band at 692 cm^–1^ corresponds to the axial stretching vibration of the C–S bond.^41,42^

Figure 2e,f show the ATR-FTIR spectra of the microspheres prepared from the precursor materials. The bands in the region of 3380 cm^–1^ in both spectra correspond to the vibrational mode of the O–H bond, while the bands observed at 1625 cm^–1^ are attributed to the symmetric and asymmetric stretching of the COO^–^ groups of the carboxylate groups present in the alginate structure. In the region of 3620 cm^–1^, bands corresponding to O–H stretching vibrations are also observed. The bands at 794 cm^–1^ and 520 cm^–1^ refer to the vibrations of the Si–O–Si and Si–O–Al bonds, respectively, present in the structure of sodium bentonite. The absence of bands corresponding to thiamethoxam in the prepared microspheres is possibly due to the low concentration of this compound in the microspheres, which compromises its identification in the spectrum.

The TGA–DTG curves of the control and thiamethoxam-containing beads are shown in Figure 3. The first mass-loss region, from room temperature to approximately 200 °C, is associated mainly with the loss of physically adsorbed and hydration water; the observed mass losses were 16% for the control beads and 12% for the thiamethoxam-containing beads. Between approximately 200 and 550 °C, overlapping thermal processes associated with the organic matrix and clay dehydroxylation occurred. Above 550 °C, further transformations led to residual masses of 51.9 and 61.6%, respectively, at 1000 °C. Because TGA–DTG curves of the individual precursors were not obtained, the overlapping thermal events cannot be assigned unambiguously, and no thermal event is attributed specifically to thiamethoxam.

**Figure 3:**
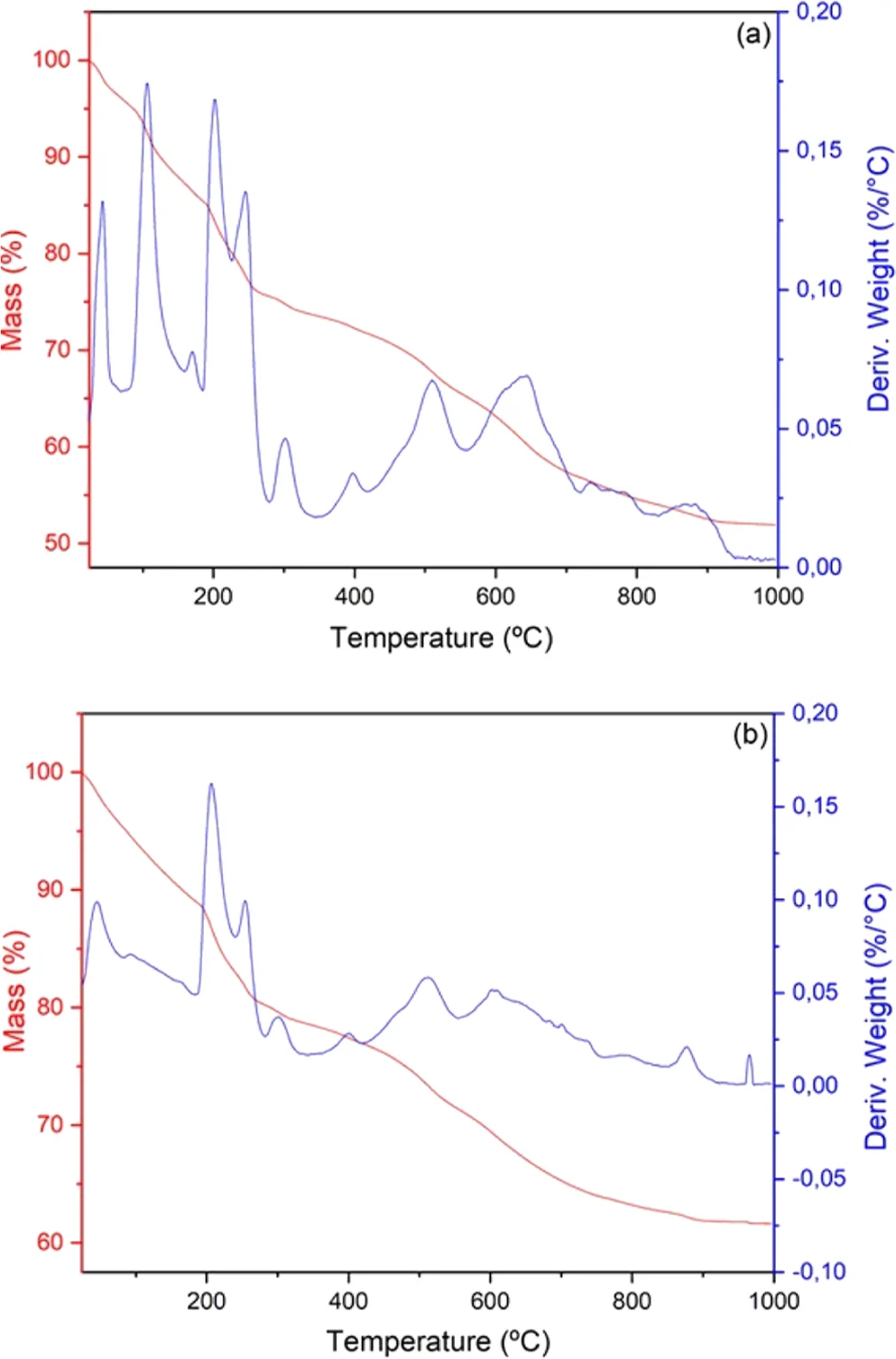
Thermogravimetric analysis (TGA) and derivative thermogravimetry (DTG) curves for (a) control beads and (b) thiamethoxam-containing beads.

The SEM images provide morphological information on the precursor materials and the prepared beads (Figure 4). The representative images indicate bead dimensions on the order of approximately 2 mm and show surface irregularities and morphological changes associated with drying. A systematic particle-size distribution was not measured; therefore, the approximate dimension should not be interpreted as a population mean or size range. SEM alone did not establish the chemical identity, spatial distribution, coating, or interaction of alginate and bentonite within the beads.

**Figure 4:**
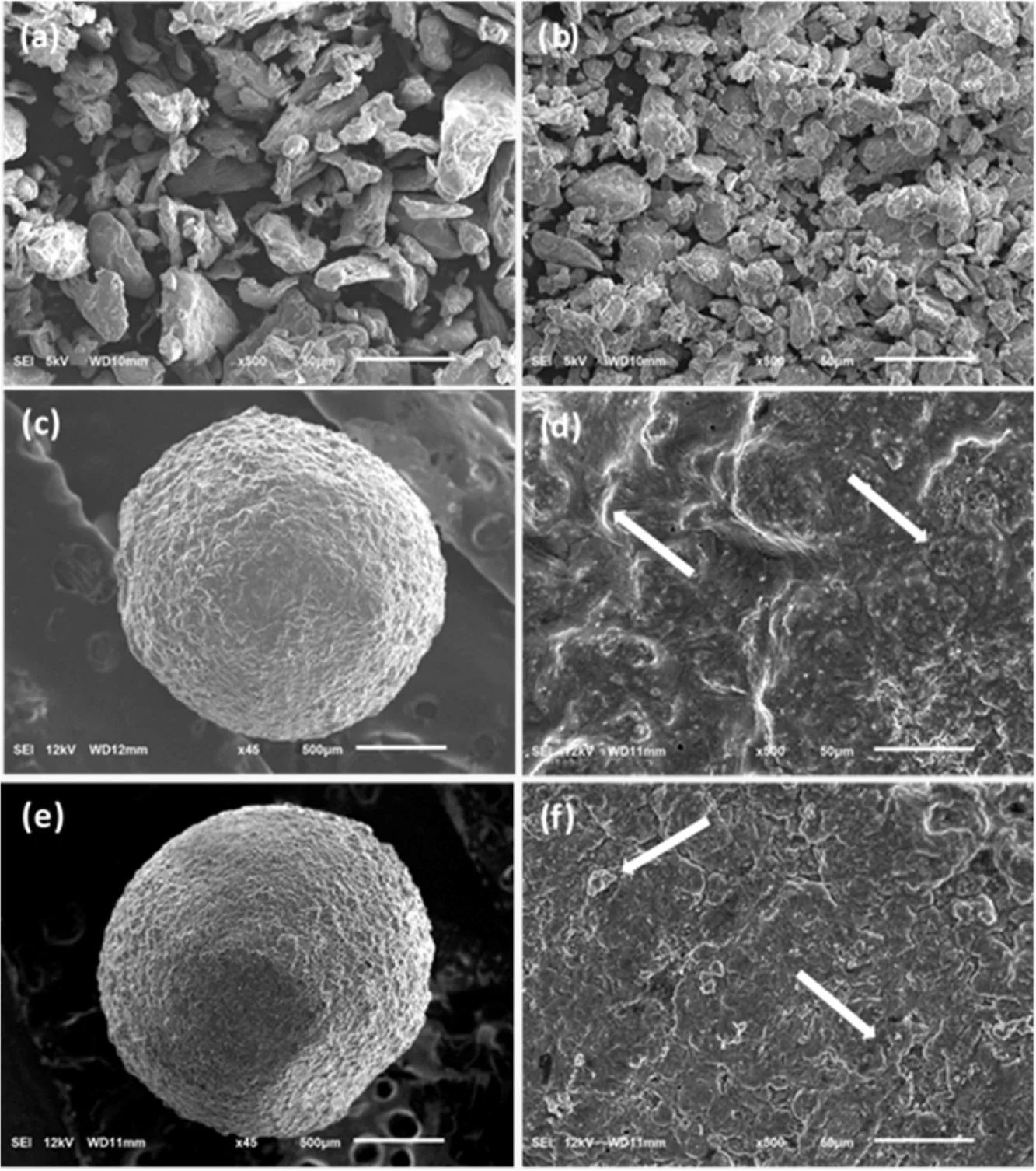
SEM images of the surface morphology of (a) sodium alginate, (b) sodium bentonite, (c,d) control beads, and (e,f) thiamethoxam-containing beads.

Figure 5 presents the baseline-adjusted fraction of the initially applied thiamethoxam recovered from the aqueous medium at each sampling time. At 0.25 h, the recovered fractions were 51.41 ± 0.27% for the alginate–bentonite bead formulation and 96.46 ± 0.12% for Actara 250 WG. At 48 h, the corresponding values were 85.71 ± 0.12% and 99.50 ± 0.04%, respectively. The bead formulation showed a lower recovered fraction than the commercial formulation over the documented 48 h interval. Because the experiment used destructive sampling, the values are not cumulative sums of serial aliquots. The bead profile was not strictly monotonic, and no kinetic or mechanistic model was assigned.

**Figure 5:**
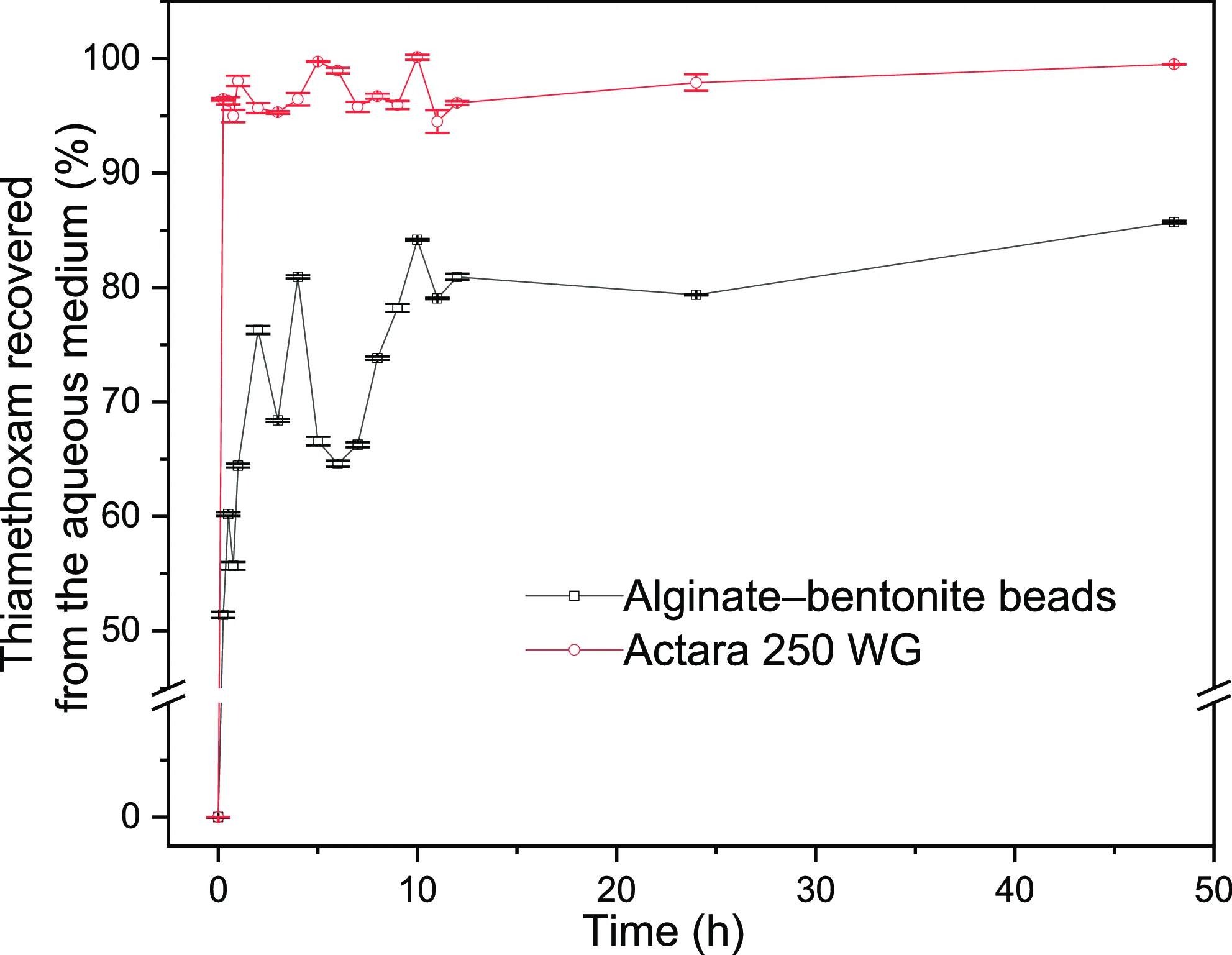
Baseline-adjusted fraction of the initially applied thiamethoxam recovered from the aqueous medium at each sampling time for the alginate–bentonite bead formulation and Actara 250 WG. Points representing standard deviations from three archived replicate responses. Connecting lines are included only as guides to visualization; no kinetic or mechanistic release model was fitted.

The aqueous profile may reflect contributions from more than one transport process. The substantial fraction recovered during the initial sampling period may be associated with rapid dissolution or desorption of thiamethoxam located at or near the bead surface or in readily accessible regions. Water penetration subsequently hydrates the Ca^2+^-cross-linked alginate network, and matrix hydration or swelling may facilitate dissolution and diffusion of thiamethoxam through water-filled pathways. The bentonite platelets may increase the tortuosity of these pathways and influence the mobility of thiamethoxam within the hydrated matrix, potentially contributing to the slower increase in the fraction recovered from the bead formulation relative to the commercial formulation. Similar general relationships among matrix compactness, water penetration, swelling, and diffusion have been discussed for other agricultural delivery systems.^43^ However, the present study did not directly determine bead swelling, diffusion coefficients, thiamethoxam distribution, matrix–thiamethoxam interactions, residual thiamethoxam after the assay, or transformation products. Therefore, this interpretation is qualitative, and no specific release mechanism is assigned.

The aqueous assay was conducted at a single buffered pH and therefore does not establish how the formulation would behave across different soil and rhizosphere pH conditions. Additional experiments at different pH values would be required to evaluate pH-dependent release. Residual thiamethoxam in the beads was not measured after the documented 48 h interval, and the experiment was not continued until equilibrium. Consequently, the unrecovered fraction cannot be assigned specifically to subsequent controlled release.

The cumulative fraction of initially applied thiamethoxam recovered from the soil-column leachate was lower for the alginate–bentonite bead formulation than for Actara 250 WG at each collection event (Figure 6). The mean fractions for the bead formulation and Actara 250 WG were 6.98 ± 1.64% and 10.04 ± 1.00% on day 1, 34.40 ± 1.74% and 52.90 ± 1.21% on day 5, and 81.13 ± 1.12% and 94.15 ± 6.91% on day 30, respectively (*n* = 4). These comparisons are descriptive because the formulations did not represent equivalent thiamethoxam amounts.

**Figure 6:**
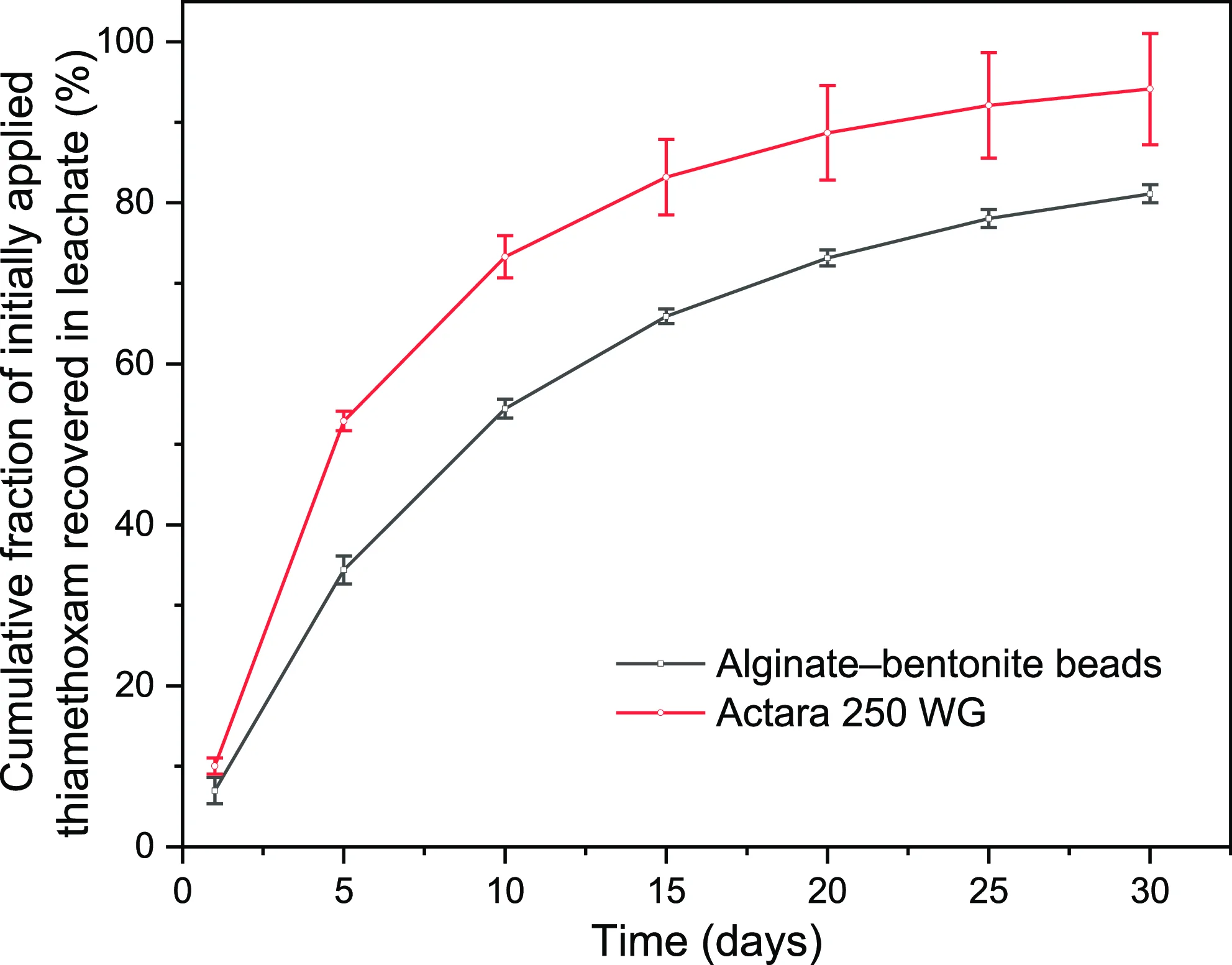
Cumulative fraction of the initially applied thiamethoxam recovered from the soil-column leachate for the alginate–bentonite bead formulation and Actara 250 WG. Points represent means ± standard deviations (*n* = 4). Calculations used the individual formulation mass and recovered leachate volume for each column. Connecting segments are included only as guides to visualization.

Only thiamethoxam recovered in the leachate was quantified. Residual thiamethoxam in the soil and beads and potential transformation products were not measured; therefore, a complete mass balance was not established. The fraction not recovered from the leachate cannot be assigned specifically to retention, degradation, incomplete extraction, irreversible association, or another process. Consequently, the lower mean leachate recovery for the bead formulation does not establish groundwater protection, reduced degradation, reduced environmental contamination, or superior environmental performance.

Both the thiamethoxam-containing bead treatment and Actara 250 WG produced high mean aphid mortality at the 0-, 7-, and 14-day challenge times (Figure 7). These results are interpreted within each assessment date only and should not be interpreted as evidence of statistical equivalence, noninferiority, prolonged control, or equivalent formulation efficiency. The formulations were not applied at equivalent thiamethoxam amounts.

**Figure 7:**
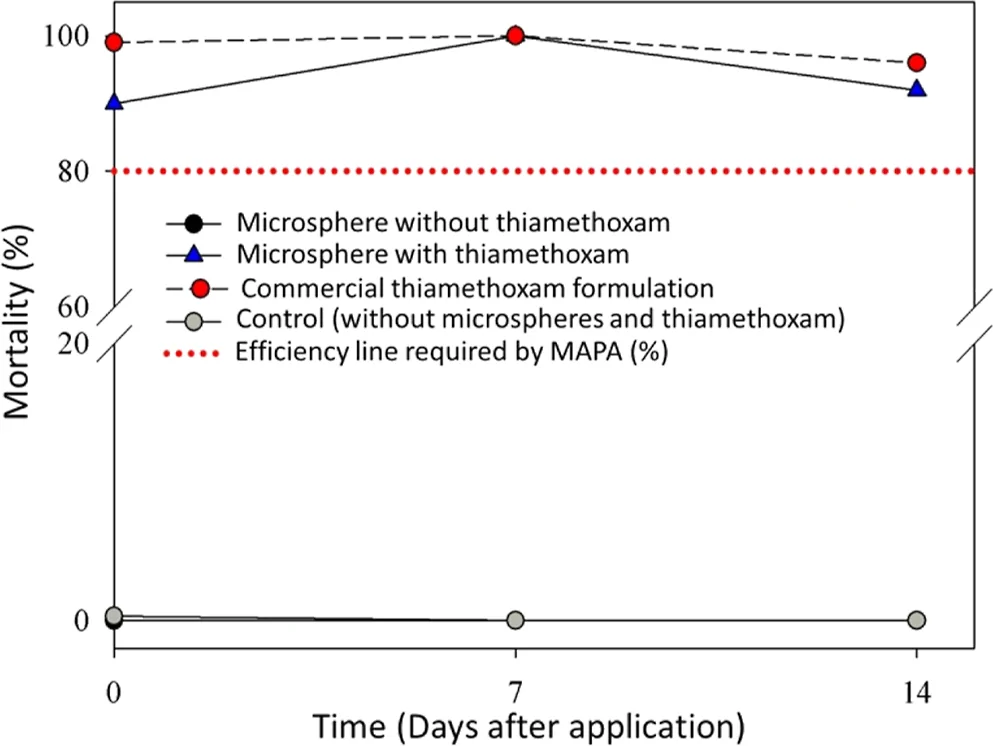
Archived mean mortality (%) of *Brevicoryne brassicae* in the four soil treatments at the 0-, 7-, and 14 day challenge times. Mortality was assessed 24 h after each infestation.

The most severe visible damage to cabbage seedlings caused by *B. brassicae* was observed in the treatment with beads without thiamethoxam and in the untreated control. Representative photographs of cabbage seedlings from the four bioassay treatments are provided in Figure S4 of the Supporting Information.

Thiamethoxam acts as a competitive modulator of insect nicotinic acetylcholine receptors. Binding to the acetylcholine site disrupts cholinergic neurotransmission and may cause hyperexcitation, lethargy, paralysis, and death. Thiamethoxam is not an acetylcholinesterase inhibitor. Thiamethoxam concentrations were not measured in cabbage tissues; therefore, aphid mortality cannot be used to quantify plant uptake or tissue concentrations.

Considering their controlled release properties, the thiamethoxam-loaded microspheres demonstrated the potential to reduce the population of *B. brassicae*. The concentration of the insecticide released was slow enough to avoid losses caused by leaching and volatilization, yet fast enough for plant uptake to kill the insects. This indicates that the synthesized beads showed similar insecticidal activity to the commercial formulation against *B. brassicae* after 7 days under the tested conditions, due to the prolonged action of the active ingredient at concentrations lethal to the insect.^41,44^

## 4 Conclusions

Alginate–bentonite beads containing extractable thiamethoxam were prepared and evaluated by physicochemical characterization, a destructive-sampling aqueous assay, a soil-column experiment, and a bioassay against *B. brassicae*. Reanalysis of the bead extracts gave an extractable thiamethoxam content of 1.219 ± 0.049 mg g^–1^. In the aqueous assay, the fraction recovered from the medium was lower for the bead formulation than for Actara 250 WG throughout the 48 h evaluation. Recalculation of the recovered soil-column records (*n* = 4) gave cumulative fractions of 81.13 ± 1.12% for the bead formulation and 94.15 ± 6.91% for Actara 250 WG on day 30. However, only thiamethoxam in the leachate was measured; residual thiamethoxam in the soil and beads and potential transformation products were not quantified, and a complete mass balance was not established. The bead treatment produced high mean mortality of *B. brassicae* at the evaluated challenge times. Nevertheless, the formulations were not compared at equivalent thiamethoxam amounts, and the study did not include a validated matrix-recovery experiment, a formal repeated-measures analysis of the bioassay, or equivalence or noninferiority testing. Therefore, the results demonstrate different behavior under the tested conditions but do not establish superior performance, prolonged insect control, groundwater protection, or reduced environmental impact.

## Supplementary Material



## Data Availability

The archived HPLC workbooks supporting the extractable-thiamethoxam-content, aqueous-release, and soil-column recalculations are available from the corresponding author upon reasonable request.

## Notes

Photographs of control beads before and after drying (Figure S1); photographs of thiamethoxam-containing beads before and after drying (Figure S2); experimental setup for the soil-column leaching assay (Figure S3); photographs of cabbage seedlings from the bioassay treatments (Figure S4); *B. brassicae* injuries on cabbage seedlings (Figure S5) (PDF)
