# Supplementary material for: Alginate–Bentonite Beads Containing Thiamethoxam: Preparation, Characterization, and Activity against Brevicoryne brassicae
Source: ACS Omega. 2026 Sep 17;11(38):57684–92. doi: 10.1021/acsomega.6c06825 (PMC13628989; doi:10.1021/acsomega.6c06825)
Supplement: Supplementary file 1 [file ao6c06825_si_001.pdf]

SUPPORTING INFORMATION

Alginate–Bentonite Beads Containing Thiamethoxam: Preparation, Characterization, and Activity against *Brevicoryne brassicae*

Silas J. de Souza[1]; Gustavo F. de Castro[2]; Jader A. Ferreira[1]; Renê C. da Silva[2]; Carlos G. da Cruz[3]; Flávio L. Fernandes[3]; Jairo Tronto[1]

[1]Instituto de Ciências Exatas e Tecnológicas, Universidade Federal de Viçosa – UFV, Rio Paranaíba, 38810-000 MG, Brazil.

[2]Departamento de Agronomia, Universidade Federal de Viçosa – UFV, Viçosa, 36570-900 MG, Brazil.

[3]Instituto de Ciências Agrárias, Universidade Federal de Viçosa – UFV, Rio Paranaíba, 38810-000 MG, Brazil.

*Corresponding author: jairotronto@ufv.br

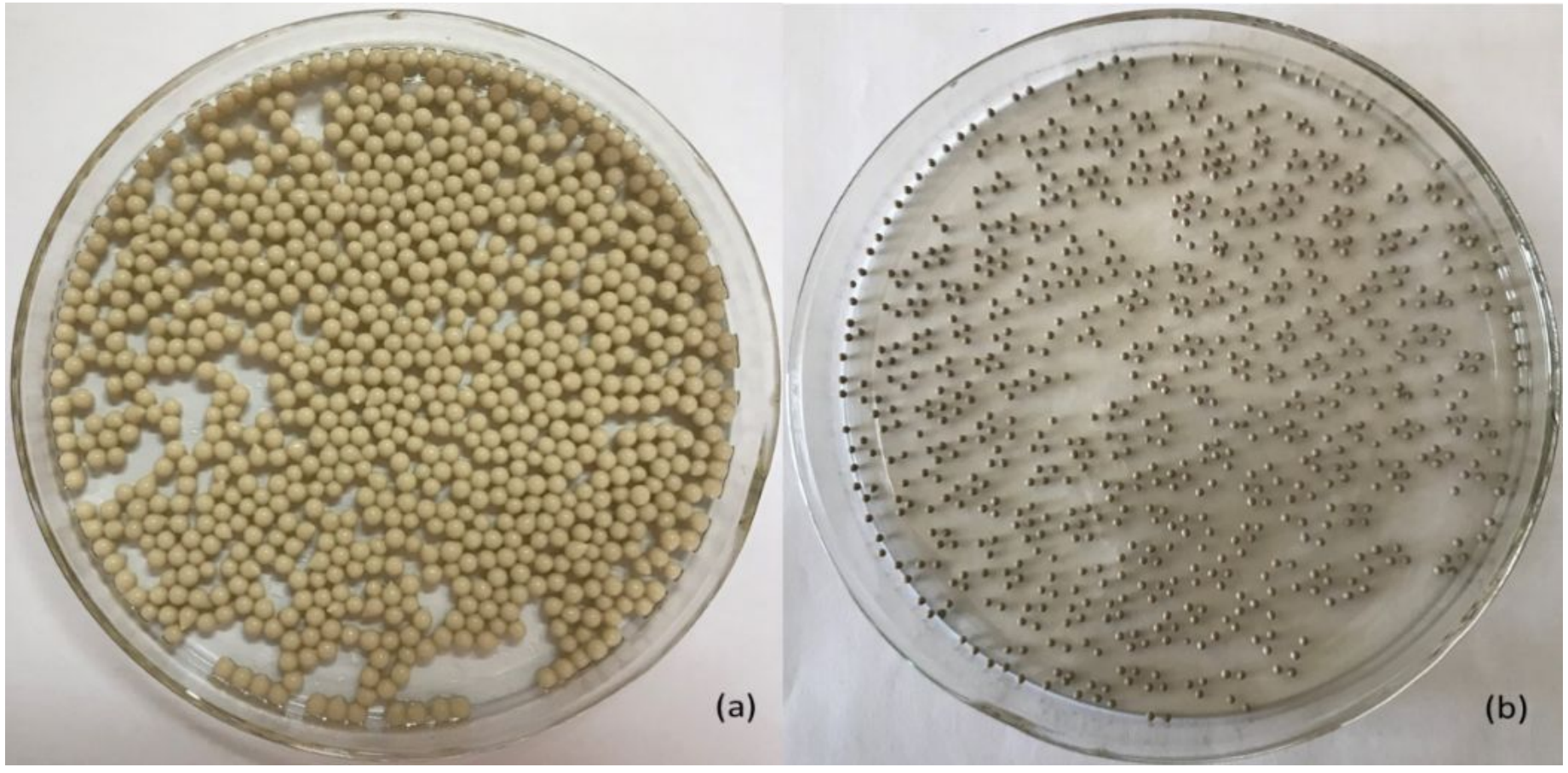

Figure S1. Photographs of the control beads (a) immediately after preparation and (b) after drying for 24 h at 40 °C.

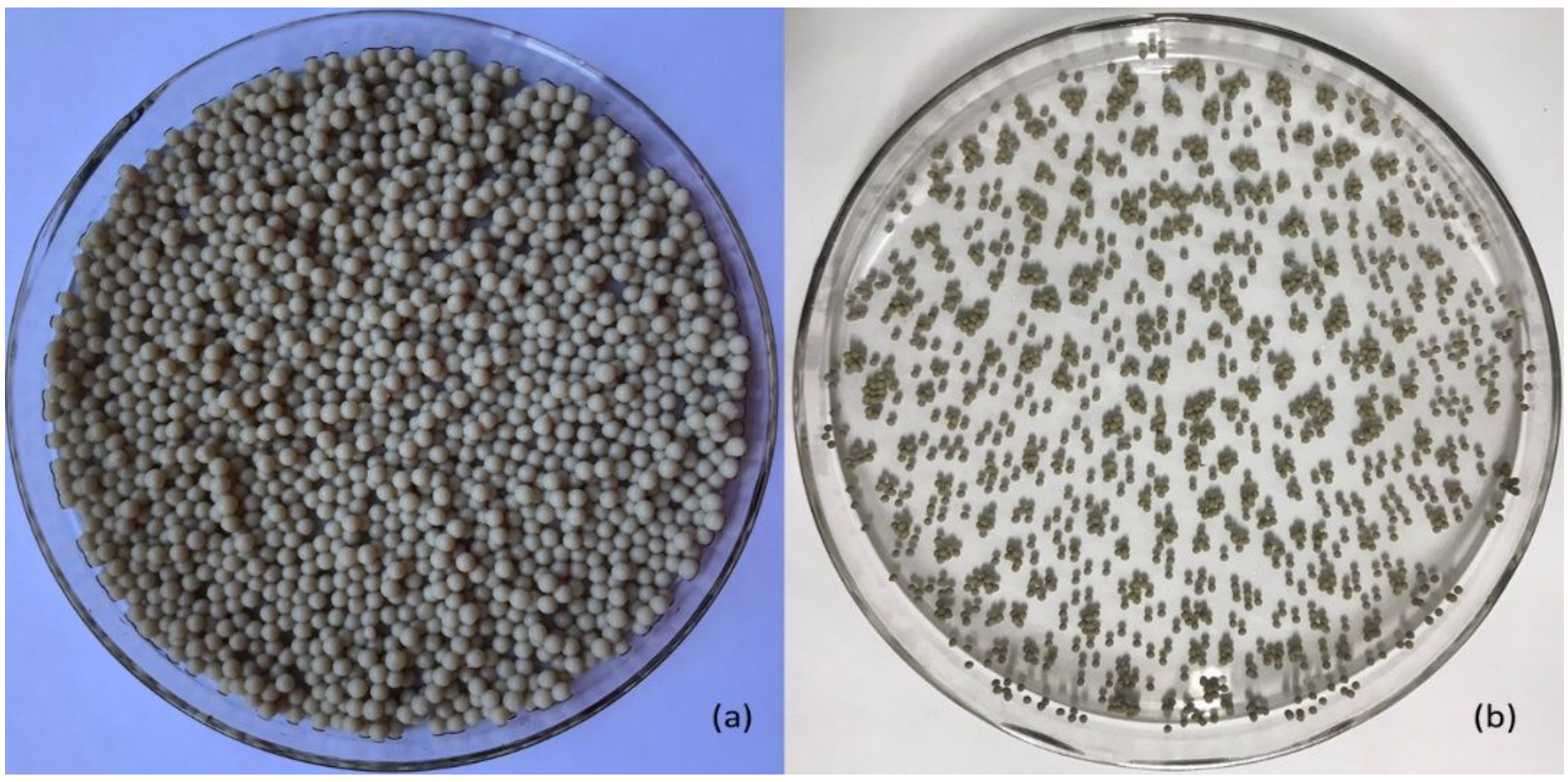

Figure S2. Photographs of the thiamethoxam-containing beads (a) immediately after preparation and (b) after drying for 24 h at 40 °C.

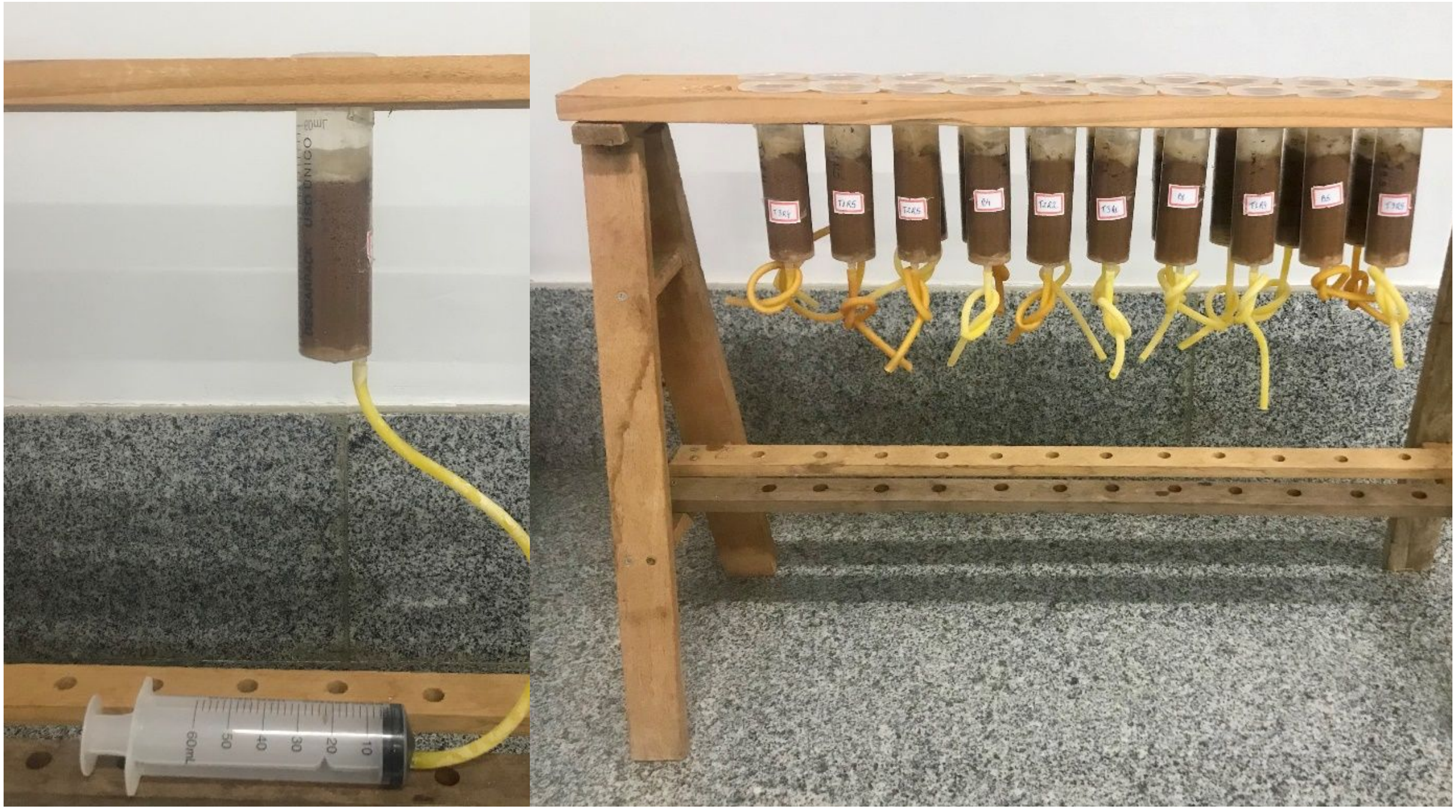

Figure S3. Experimental setup used for the soil-column leaching assay.

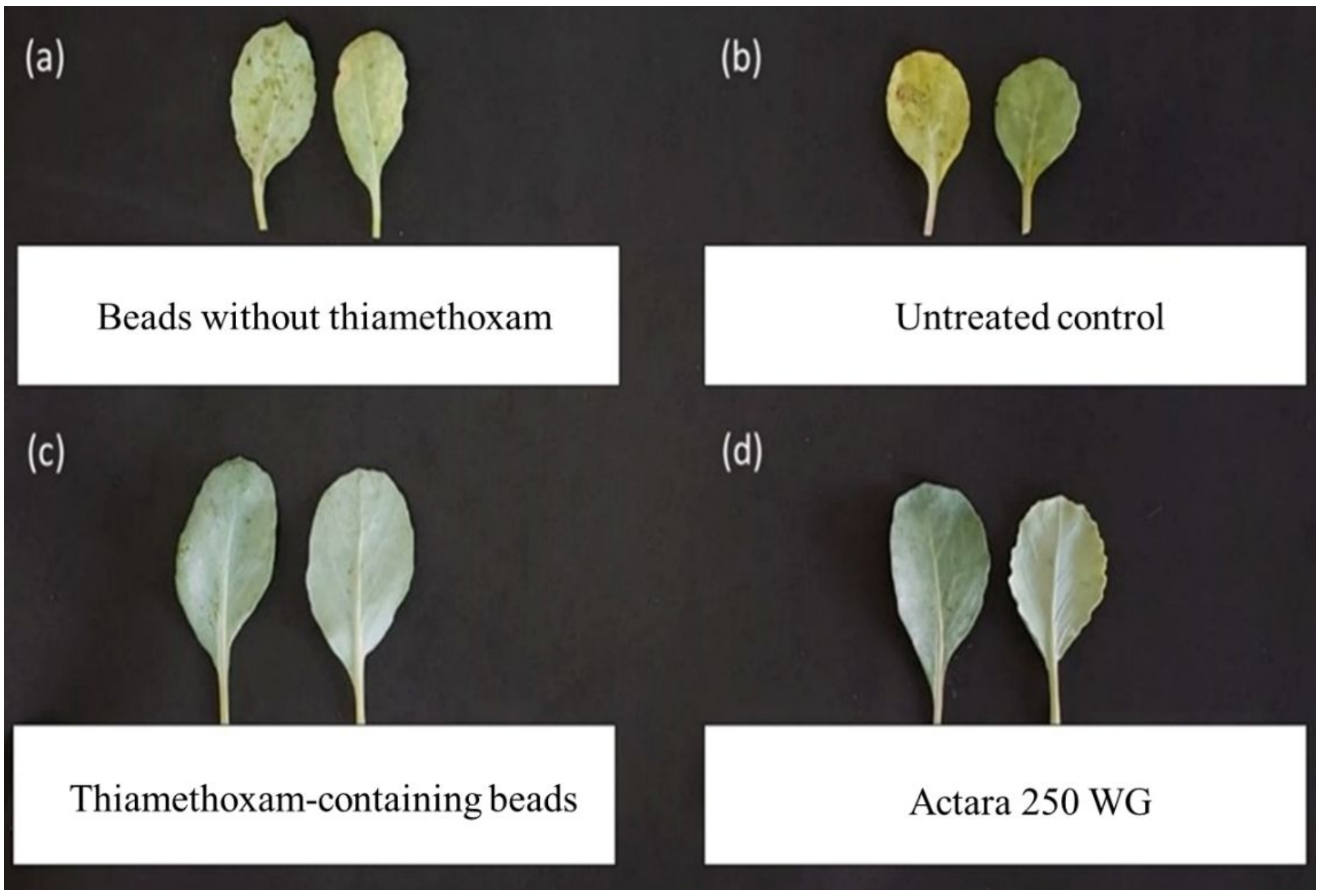

Figure S4. Cabbage seedlings from the bioassay treatments: (a) beads without thiamethoxam, (b) untreated control, (c) thiamethoxam-containing beads, and (d) Actara 250 WG.

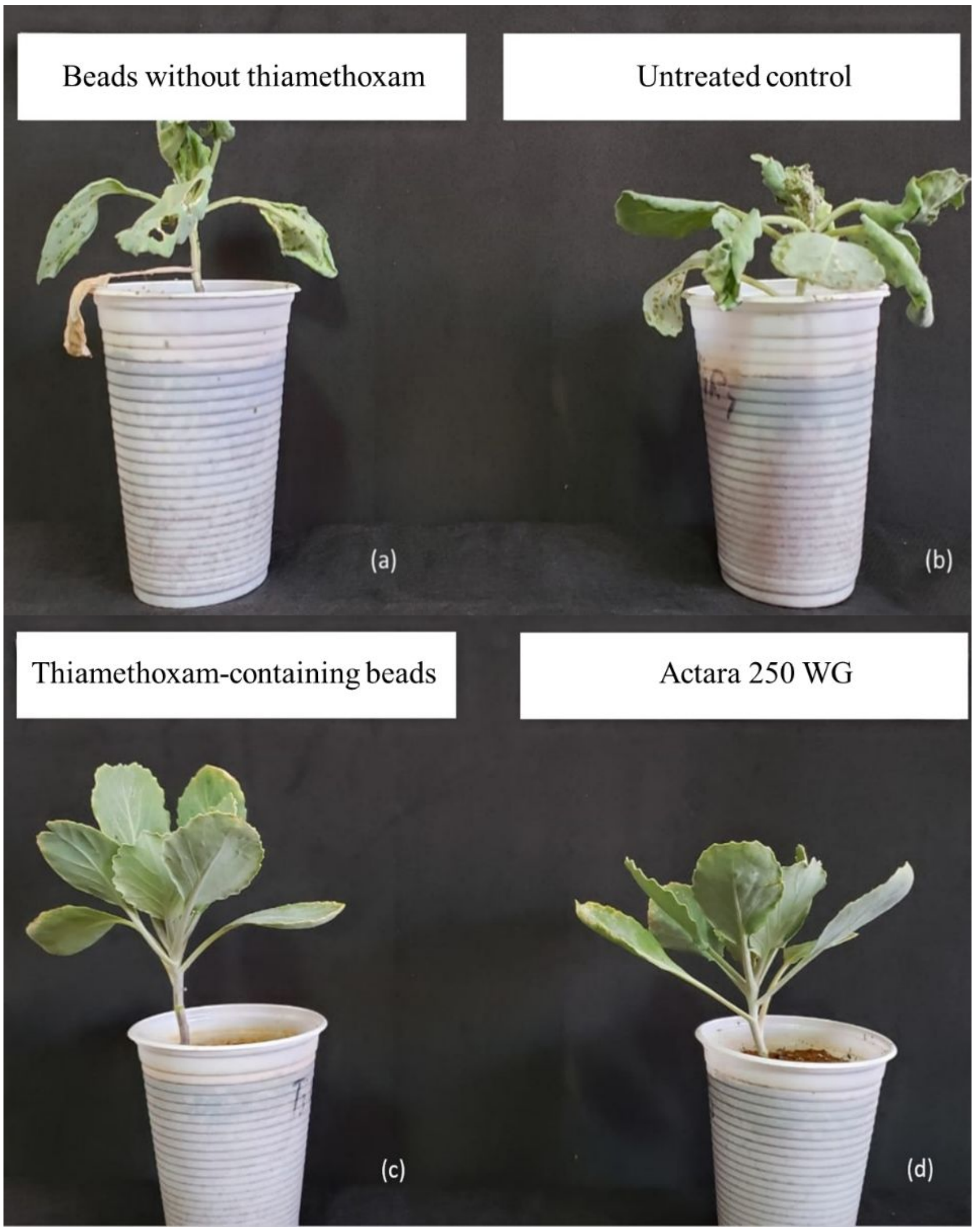

Figure S5. *B. brassicae* injuries on cabbage seedlings in treatments: (a) beads without thiamethoxam; (b) untreated control; (c) thiamethoxam-containing beads; and (d) Actara 250 WG.

34
